# Regulation and function of transposable elements in cancer genomes

**DOI:** 10.1007/s00018-024-05195-2

**Published:** 2024-03-31

**Authors:** Michael Lee, Syed Farhan Ahmad, Jian Xu

**Affiliations:** 1 Department of Pediatrics, Children’s Medical Center Research Institute, University of Texas Southwestern Medical Center, 6000 Harry Hines Blvd., Dallas, TX 75390 USA; 2 Department of Pathology, Center of Excellence for Leukemia Studies, St. Jude Children’s Research Hospital, 262 Danny Thomas Place – MS 345, Memphis, TN 38105 USA

**Keywords:** Non-coding genome, Retrotransposons, LINE-1, SINE, ERVs, Viral mimicry, Long-read sequencing

## Abstract

Over half of human genomic DNA is composed of repetitive sequences generated throughout evolution by prolific mobile genetic parasites called transposable elements (TEs). Long disregarded as “junk” or “selfish” DNA, TEs are increasingly recognized as formative elements in genome evolution, wired intimately into the structure and function of the human genome. Advances in sequencing technologies and computational methods have ushered in an era of unprecedented insight into how TE activity impacts human biology in health and disease. Here we discuss the current views on how TEs have shaped the regulatory landscape of the human genome, how TE activity is implicated in human cancers, and how recent findings motivate novel strategies to leverage TE activity for improved cancer therapy. Given the crucial role of methodological advances in TE biology, we pair our conceptual discussions with an in-depth review of the inherent technical challenges in studying repeats, specifically related to structural variation, expression analyses, and chromatin regulation. Lastly, we provide a catalog of existing and emerging assays and bioinformatic software that altogether are enabling the most sophisticated and comprehensive investigations yet into the regulation and function of interspersed repeats in cancer genomes.

## Introduction

Transposable elements (TE) or “transposons” are mobile DNA parasites that can change their chromosomal positions within a genome through a molecular process called transposition. By virtue of their mobility, TEs have widely colonized genomes throughout life—coevolving with their host organisms—and in many cases make up significant fractions of their host genome [1]. In humans, TEs compose over half of genomic DNA [2, 3], interspersed between and within protein-coding genes as full-length or truncated copies. Despite their abundance, TEs have historically been viewed as “junk” or “selfish” DNA inconsequential to the phenotype of their hosts [4], in part due to the lack of tools to test alternative hypotheses. Over the past two decades, advances in sequencing and computational methods have overcome many longstanding challenges in studying TEs and, increasingly, are revealing their central roles in genome regulation and evolution, bringing to life Barbara McClintock’s prescient “controlling elements” hypothesis [5].

Equally important progress is underway to unravel precisely how TEs contribute to human diseases including cancer. Recent studies have uncovered seemingly contradictory roles of TEs in cancer, wherein their activity has been linked to both cancer promoting and suppressive functions, suggesting that the regulation and impact of TEs in cancer are highly context- and cell type-specific. Efforts to discern the mechanistic basis of this dichotomy are informing how TE biology could be rationally exploited for improved anti-cancer therapies. We begin this review with a general overview of the landscape of TEs within the human genome. We then discuss the current views on how the various activities of TEs have been implicated in cancer biology and treatment. Lastly, we describe the longstanding challenges in studying interspersed repeats and how new experimental and computational tools, particularly those based on long-read sequencing, are rapidly improving our abilities to catalog de novo TE content in genomes and to mechanistically dissect their contribution to cancer development.

## Human transposable elements

TEs have undergone waves of expansion and decay throughout mammalian evolution and primate speciation such that present-day human genomes host diverse TE families and their sequence remnants [1, 6]. Distributed irregularly throughout genomic DNA, TEs are considered “interspersed” repeats to distinguish them from “tandem” repeats, which are repetitive DNA that compose much of the telomeric and centromeric regions of human chromosomes (reviewed in [7, 8]). TEs can be broadly classified into two major taxonomic groups based on their biochemical modes of transposition: type I TEs or “retrotransposons” and type II TEs or “DNA transposons”.

**Fig. 1:**
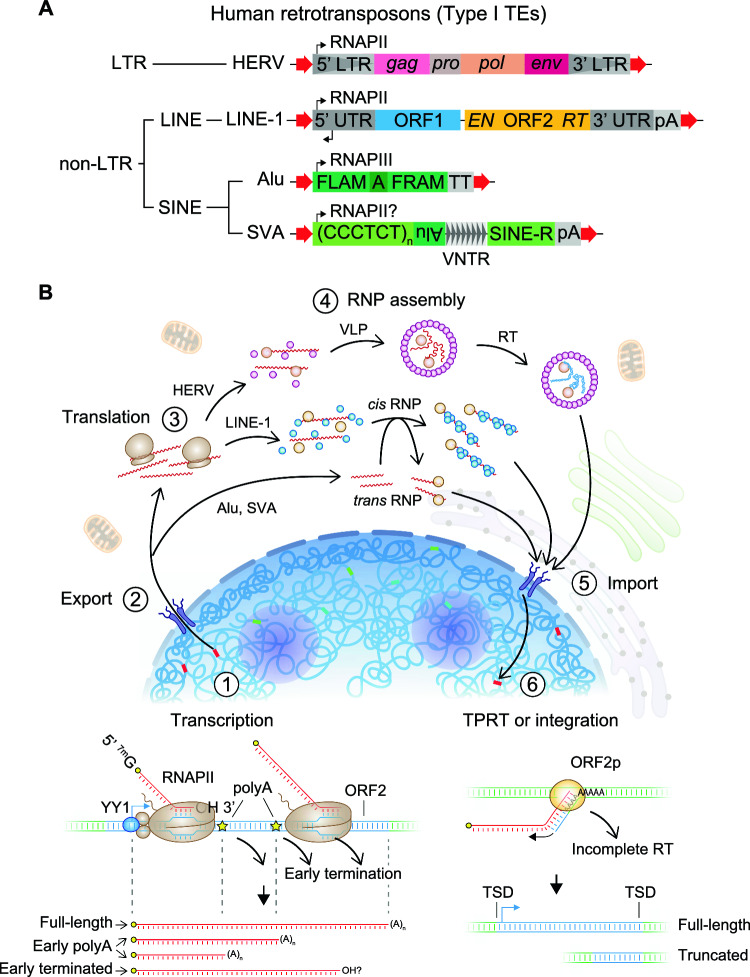
Human retrotransposons and their replication cycle. **A** Domain schematics of the major retrotransposons in the human genome. Thick red arrows depict target site duplications (TSD), a hallmark of retrotransposition. Thin black arrows depict transcription start sites. LINE-1 5′UTR possesses an anti-sense promoter. SVA elements most likely are transcribed by RNAPII. LTR, long terminal repeat. UTR, untranslated region. RNAPII, RNA polymerase II. FLAM, Free Left *Alu* Monomer. FRAM, Free Right *Alu* Monomer. VNTR, variable tandem repeat. pA, poly-adenylation signal. TT, T-stretch terminator of RNAPIII. **B** Key steps of the retrotransposition cycle for LINE-1, Alu/SVA, and HERV. Alu and SVA are non-autonomous and hijack LINE-1 machinery in *trans* for TPRT. The HERV RT reaction occurs within virus-like particles (VLP) prior to nuclear import and integration. LINE-1-mediated TPRT preferentially targets AT-rich sequences. Major RNA species of LINE-1 are depicted below its transcription reaction schematic. YY1 positions proper LINE-1 TSS selection. 5′-^7m^G denotes the 7-methylguanosine cap of LINE-1 mRNA. RNP, ribonucleoprotein particle. RT, reverse transcription. TPRT, target-primed reverse transcription

Retrotransposons, so-called because of their “copy-and-paste” replication strategy defined by reverse transcription, are the predominant TEs in humans and contain various subfamilies still actively proliferating. In contrast, DNA transposons, which mobilize via a “cut-and-paste” excision-reinsertion mechanism using self-encoded transposases, are evolutionarily older and largely immobile in humans. Type I TEs can be further classified into “LTR” versus “non-LTR” retrotransposons depending on their sequence structure as well as “autonomous” versus “non-autonomous” depending on whether they encode the enzymatic machinery sufficient to replicate themselves (Fig. 1A). Long terminal repeat (LTR) retrotransposons comprise diverse lineages of endogenous retroviruses that are expressed but likely no longer transpose in humans [9]. Non-LTR retrotransposons include Long Interspersed Elements (LINEs) and Short Interspersed Elements (SINEs). In this section, we introduce the major retrotransposons of the human “mobilome” and the general features of their replication cycle. We also discuss the current views on how TEs have uniquely been domesticated as regulators of genome structure and function.

### Human endogenous retroviruses

Retroviruses have infected vertebrate genomes for hundreds of millions of years, representing one of the oldest forms of infection [10]. Unlike other infectious agents, retroviruses are particularly pernicious in that their replication strategy entails integration into host genomic DNA, allowing for indefinite propagation to all soma as well as future generations of an organism upon successful invasion of the germline. Endogenous retroviruses (ERVs) are thought to originate from ancient retroviral germline integrants that subsequently lost, likely by accumulating mutations, the capacity to produce exogenous virion while retaining the enzymatic capacity for integration, thus becoming a de facto retrotransposon [10]. Like their retroviral counterparts, ERVs encode *gag*, *pro*, *pol*, and sometimes *env* open reading frames (ORF) flanked by their namesake regulatory sequences called long terminal repeats (LTR). The *gag* gene product scaffolds the proteinaceous “capsid” that protects the ERV RNA genome along with *pol* gene products within a so-called virus-like particle (VLP) during ERV transposition. The *pol* ORF encodes a reverse transcriptase (RT) with an RNase H domain and an integrase (INT). Once encapsulated, the VLP is imported into the nucleus, sheds the reverse-transcribed complementary DNA (cDNA) ERV genome bound at their ends by INT molecules, and, finally, the cDNA provirus is integrated into host genomic DNA via INT catalysis (Fig. 1B).

While human ERVs (HERV) are known to be expressed and can produce VLPs, there is currently no evidence of active retrotransposition by extant HERVs [9]. This contrasts with the situation in other mammals, such as mice, where many ERV subfamilies are still actively transposing [11]; the compositional difference in active TEs between mice and humans is worthy of consideration when reconciling various experimental findings between the two species. Interestingly, the most recently acquired HERV family, HERV-K, which includes the youngest human-specific subfamily, HML-2, has loci that are polymorphic in humans, *i.e.*, present in some individuals but absent in others, suggesting that HML-2 may have been active in the recent past [9]. Nevertheless, most ERV subfamilies in humans are immobile, existing as either proviral forms or solitary LTRs formed by intra-element homologous recombination resulting in deletion of the internal coding sequence [9].

### LINE-1 retrotransposons

The LINE-1 retrotransposon family contains the only elements in humans that can still autonomously transpose [12]. Most of the ~ 500,000 LINE-1 copies in the human reference genome are truncated or mutated, leaving ~ 100 source alleles that are full-length and competent for transposition [13, 14]. Current estimates are that LINE-1 insertions occur once in every ~ 63 live births [15]; thus, LINE-1 sequences are highly polymorphic among human populations. Retrotransposition events are generated by intact LINE-1 elements ~ 6 kb in length, encoding two ORFs flanked by 5′ and 3′ untranslated regions (UTR). LINE-1 transcription is driven by an internal RNA polymerase II (RNAPII) promoter within the 5′ UTR [16]. The 5′ UTR promoter encodes various transcription factor (TF) binding motifs including SOX family TFs which contribute to the cell type-specificity of LINE1 expression [17]. YY1 is also known to bind at a motif on the antisense strand between position + 21 to + 13 to guide the fidelity of transcription start site selection and ensure propagation of the 5′UTR promoter [18]. Upon initiation, the RNAPII complex elongates through ORF1 and ORF2 and typically terminates at a polyadenylation (polyA) signal within its 3′UTR (Fig. 1B). Curiously, transcription elongation frequently stalls within ORF2 due to high AT content (~ 67%) or cryptic polyA signals [19, 20]. These sequence features may have evolved to limit LINE-1 retrotransposition [19–21].

When a full-length LINE-1 transcript does form, the mRNA is subsequently exported to the cytoplasm, likely mediated by an NXF1 recognition motif within its 3′UTR, where it undergoes ribosomal translation [22]. The ~ 40 kDa ORF1 gene product (ORF1p) has RNA binding and nucleic acid chaperone activities [23], although its exact function remains incompletely understood. The ~ 150 kDa ORF2p protein encodes endonuclease and reverse transcriptase domains [24, 25]. Importantly, both ORF1p and ORF2p are required for retrotransposition; however, how the stoichiometry of human ORF1p and ORF2p translation is regulated is not known [26]. ORF1p and ORF2p binds to LINE-1 mRNA preferentially *in cis* [27, 28], assembling into ribonucleoprotein particles (RNPs) that are imported into the nucleus. Finally, genomic integration occurs following ORF2p-mediated nicking of genomic DNA at AT-rich sequences and reverse transcription which occurs directly at the target locus, a unique replication mechanism of non-LTR retrotransposons termed “Target Primed Reverse Transcription” (TPRT). Notably, for still unclear reasons, TPRT is error-prone and reverse transcription often prematurely terminates generating the many 5′ truncated insertions found throughout the genome [12] (Fig. 1B).

### Alu and SVA elements

Alu and SVA elements are the major constituents of the SINE family in the human genome. Alu elements are among the most prolific human mobile elements as measured by their copy number relative to their short sequence structure of 100–300 bp, totaling ~ 11% of human genomic DNA [29]. Alu sequences are thought to have derived from an ancestral form of the *7SL RNA* gene, the non-coding RNA component of the signal recognition particle complex, at some point during primate speciation [29]. Alu elements contain two arms, the “free left Alu monomer” (FLAM) and the “free right Alu monomer” (FRAM), along with an internal RNA polymerase III promoter and a 3′ stretch of adenines called the “A-tail” (Fig. 1A). Alu transcription does not have an encoded termination signal but instead uses the most proximal TTTT terminator sequence downstream of its locus [29]. While convergently evolved Alu relatives exist in rodents, such as the B1 SINEs in mice, the SVA element is a hominid-specific composite retrotransposon [30], made up by the fusion of three major repeat components, namely SINE-R (homologous to a HERV), a variable number tandem repeat (VNTR) segment, and an Alu-like region. Importantly, Alu and SVA elements are both non-autonomous TEs and coopt the LINE-1 ORF2p machinery in *trans* for their replication by TPRT. Both Alu and SVA elements are highly polymorphic, with approximately one Alu insert per ~ 40 human births and one SVA insertion per ~ 60 human births [15, 29]. SVA elements have been less studied due to the difficulty of resolving their composite repeat structure by conventional short-read-based sequencing; however, recent studies demonstrate that long-read sequencing technologies enable comprehensive cataloging of polymorphic SVAs across diverse human populations [31].

### TEs as genome regulators

**Fig. 2:**
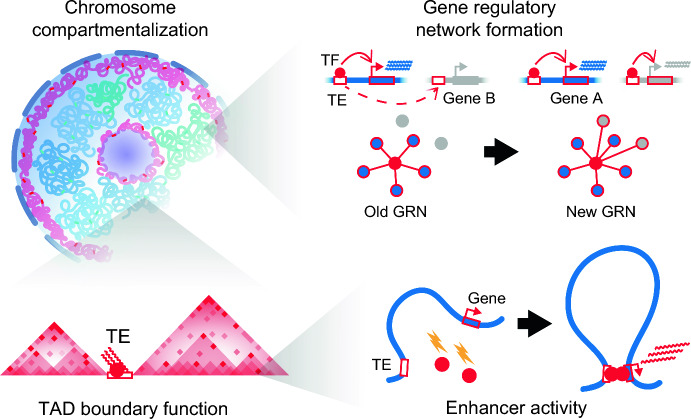
TE activity generates genomic variation and is coopted during evolution. TE activity has frequently been coopted for beneficial regulatory functions in genomes during evolution, including chromosome compartmentalization, TAD boundary formation, enhancer activity, and gene regulatory network formation. GRN, gene regulatory network. TF, transcription factor. Dashed line with arrowhead depicts a transposition event. Lightning symbol depicts signaling cues such as cytokines triggering TF binding and activating interferon-stimulated gene (ISG) transcription

Barbara McClintock, who discovered the first TEs in maize, envisioned in the 1950s that TEs may act as fundamental “controlling elements” in genomes, dynamically regulating host gene expression to impart cellular complexity [5]. In 1979, Britten and Davidson introduced the “gene-battery” model theorizing that TEs could represent the evolutionary substrates underlying the formation of gene regulatory networks by transposing their embedded regulatory sequences throughout their host genomes [32]. Although provocative, these seminal theories remained largely untested over the ensuing quarter-century until the advent of genomic technologies in the early 2000s. Over the past decade, numerous studies have now demonstrated that TEs, particularly ERVs, have in fact frequently been coopted by host organisms and repurposed for key gene regulatory and cellular functions [6] (Fig. 2). Lynch et al. identified ancient DNA transposons of the MER20 subfamily that have recurrently installed hormone-responsive *cis*-regulatory sequences within pregnancy-related gene regulatory networks during the evolution of placental mammals [33]. Similarly, Chuong et al. discovered lineage-specific ERVs that bind interferon-induced transcription factors regulating key innate immune genes including *AIM2* [34]. Importantly, the authors leveraged CRISPR/Cas9 technology to delete specific ERVs and provided evidence that TEs can regulate host gene expression as *cis*-regulatory elements (CRE). These studies and others [6, 35–44] have substantiated the “controlling elements” and “gene battery” paradigms wherein host organisms exploit the mobility of TEs throughout evolution by repurposing TE-derived regulatory DNA distributed across their genomes into CREs, creating new gene regulatory networks (Fig. 2).

### TEs as genome architects

As eukaryotes evolved multi-cellularity, organisms had to adopt strategies to create specialized transcriptomes using the same underlying genomic blueprint. Moreover, nuclear space became increasingly limited as genomes expanded in size, in part due to the prolific copy number expansion of TEs [45]. The solution to both problems arose in the form of structural mechanisms to compact and spatially segregate interphase genomic DNA into nested three-dimensional topologies wherein functionally related genes can be co-regulated by being physically looped together in proximity to cell type-specific CREs [46]. These organizing 3D structures, termed “topologically associated domains” (TAD), are formed by the anchoring of architectural transcription factors including the 11-zinc finger CCCTC-binding factor (CTCF) at so-called TAD boundaries. Here, genomes have once again leveraged TE sequences. Through comparative epigenomic studies, Schmidt et al. uncovered that conserved species-specific CTCF binding sites are highly enriched for SINE family retroelement sequences across mammalian genomes, suggesting that retrotransposon expansion has driven the species-specific dispersal of CTCF sites throughout mammalian evolution to remodel genome structure [47].

Within species, TE-derived CTCF boundary elements may also regulate developmental and cell type-specific chromatin architecture [48]. Zhang et al. demonstrated in human pluripotent stem cells that source loci of the primate-specific HERV-H retrotransposon family bound CTCF and exhibited developmental stage-specific boundary activity [49]. CRISPR-mediated deletion of individual HERV-H elements abolished TAD structures and altered expression of the contained genes. Importantly, insertion of an HERV-H element by PiggyBac transduction of an ectopic HERV-H donor element was sufficient to establish de novo TAD structures [49], providing evidence that transposition of retroelement sequences can remodel chromatin architecture. Interestingly, the authors also identified that HERV-H transcription was required for its boundary activity, as CRISPR-mediated inhibition of the locus was sufficient to impair TAD integrity, suggesting a role for retroelement RNAs in mediating *cis*-regulatory activity. Similar findings have been observed in mouse embryonic stem cells wherein LINE-1 and B1 SINE RNAs, respectively, facilitate the compartmentalization of the mouse genome into heterochromatic and euchromatic compartments [50] (Fig. 2). Notably, the Murine Endogenous Retroviral Element (MERVL) has also been shown to remodel 3D chromatin organization through transcription-associated boundary activity during mouse early embryogenesis [51], highlighting the possibility that diverse TE sequences have repeatedly been coopted throughout evolution for genome regulation.

In summary, TEs have fundamentally shaped genomic architecture in mammals, both in structure and function, through their unique capacity to mobilize regulatory DNA. Far from their long-held misnomer as “junk”, TEs represent a mutagenic force which has enabled genomes to restructure and adapt under changing environments throughout evolution. Yet, just as TE activity can prove beneficial to organisms, so too it can promote disorder and disease, which we consider next.

## Regulation and function of transposable elements in cancer genomes

Cancer cells represent aberrant forms of their cell types of origin, characterized by the sequential acquisition of genetic changes promoting unchecked proliferation and enhanced cellular adaptation. TEs, as natural mutagens, thus seem ideal agents of change for cancers. Indeed, while transcriptionally silenced in most somatic tissues, TEs become widely reactivated during cellular transformation [52]. However, whether TE activity is a cause or consequence of cancer development remains a complex, unresolved question. In this section, we discuss the current understanding of how TEs become dysregulated in cancer and how the various intermediates of their replication cycles potentially contribute to cancer progression, with a particular focus on LINE-1 retrotransposons. We then contrast these models with recent studies revealing surprising tumor-suppressive functions of TEs in certain contexts. Lastly, we describe the conceptual basis for on-going efforts to leverage TE activity for cancer type-specific therapy.

### TE dysregulation in cancer

TE reactivation is an emerging hallmark of cancers [52, 53]. Rodić et al. performed an immunohistochemistry (IHC) survey of a diverse panel of human tumors and found that nearly half of all cancers tested were immunoreactive for LINE-1 ORF1p, with high-grade tumors highly reactive and early-stage lesions only rarely so, whereas ORF1p labeling was absent in normal somatic tissues [54]. In another study of breast cancer patients, Chen et al. identified a prognostic correlation with LINE-1 ORF1p and ORF2p expression, where tumors with higher LINE-1 protein staining by IHC were associated with more aggressive clinicopathologic features and worse patient survival [55]. Interestingly, the nuclear localization of LINE-1 protein was associated with the presence of lymph node metastases [55], suggesting that nuclear imported LINE-1 complexes may somehow promote more aggressive cancer phenotypes. Together, these studies and others [53, 56] raise the possibility that LINE-1 ORF1p expression may be a useful biomarker for cancer screening. Indeed, Taylor et al. recently introduced a proof-of-concept immunoassay for the ultra-sensitive detection of ORF1p in human plasma as a candidate tumor-specific antigen for early cancer detection, risk stratification, and treatment response monitoring of epithelial cancers [57]. Thus, LINE-1 expression is intricately linked with carcinogenesis in some cancer types.

The precise timing and exactly how TEs are reactivated in cancer remains poorly understood, although it is generally assumed to coincide with the global DNA hypomethylation characteristic of most cancer genomes [58]. The LINE-1 5′UTR promoter contains CpG dinucleotides that are frequently reported to be hypomethylated in primary tumors relative to normal tissues as well as in cell line models across diverse cancers [16, 59–61]. Most studies measure global LINE-1 methylation by PCR-based assays. Interestingly, Lanciano et al. recently devised a method to examine the methylation status of individual LINE-1 loci by high-throughput sequencing and found that LINE-1 promoter hypomethylation is heterogenous across individual source copies within and between cancer cell types; moreover, hypomethylation of a locus was not always congruent with its expression [62, 63]. Thus, while LINE-1 promoter hypomethylation is generally necessary for reactivation, it is likely not sufficient. One explanation is that multiple silencing pathways are redundantly active at LINE-1 promoters. Indeed, host organisms have evolved diverse transcriptional and post-transcriptional mechanisms to silence TEs (reviewed elsewhere [38, 64–66]). Another possibility is that cell type-specific TFs [67] and chromatin configurations permissive for their binding are required for robust LINE-1 transcription.

Taken together, the regulation of LINE-1 transcription in cancer is highly complex and dependent on both cell type- and context-specific mechanisms. Individual source loci of TEs within any given genome are likely subject to locus-specific modes of regulation and transcriptional potential based on the confluence of repressive mechanisms and TF activity in situ. Indeed, there is abundant evidence that the majority of LINE-1 retrotransposition activity derives from only a small subset of cell type-specific “hot” LINE-1 copies [13, 68, 69]. Thus, technologies to profile LINE-1 transcription and epigenetic status with locus-specific resolution will be required to elucidate the complex regulatory language governing LINE-1 expression in cancers.

### Cancer-promoting roles of TEs

**Fig. 3:**
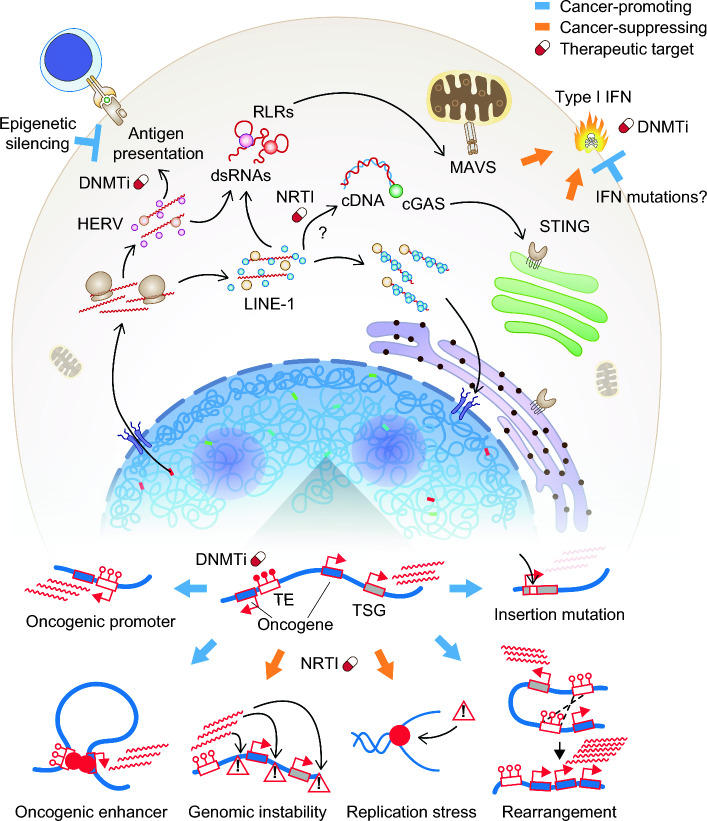
TE activity can promote and suppress cancers. Major cancer-promoting (blue arrows) and suppressive (orange arrows) roles of TEs. Antigen presentation genes are often epigenetically silenced in cancer cells to evade adaptive immunity. Some cancer types may mutate IFN related genes as an adaptive mechanism to tolerate TEs without inducing an IFN response. The mechanism of cytosolic LINE-1 cDNA synthesis is currently unknown. TSG, tumor-suppressor gene. Caution symbols depict DNA damage. RLR, RIG-I-like Receptors. dsRNA, double stranded RNA. cDNA, complementary DNA. IFN, interferon. DNMTi, DNA methyltransferase inhibitors. NRTI, nucleoside reverse transcriptase inhibitors

The disease consequence of transposition in humans was first demonstrated in 1988 by Haig Kazazian and colleagues who identified two patients with independently acquired LINE-1 insertions disrupting exon 14 of their Factor VIII gene, causing hemophilia [70]. That same year, an intronic LINE-1 sequence was identified in the *myc* locus of a patient’s breast carcinoma compared to matched normal breast tissue, implicating insertional mutation in cancer for the first time; however, the lack of sequence information of the 5′ breakpoint and the intronic position of the insertion site precluded functional interpretation [71]. A definitive case of oncogenic LINE-1 mutagenesis was described in 1992 by Miki et al. wherein they identified a ~ 790 bp LINE-1 insertion within the last exon of the *APC* tumor suppressor gene in colon cancer [72]; importantly, the shorter insert allowed the authors to retrieve both 5′ and 3′ breakpoints which revealed target site duplication (TSD), a hallmark of *bona fide* LINE-1 retrotransposition. In the years since, the number of LINE-1 insertions identified in cancers have steadily increased, including colon [69, 73], lung [74, 75], pancreas [76], ovarian [60, 77], and liver [78, 79]. To date, more than 120 cases are known of human genetic diseases and cancers caused by LINE-1 insertional mutation [80]. These early studies altogether have established the paradigm that LINE-1 retrotransposition, when it disrupts genes, is generally deleterious and may contribute to disease initiation (Fig. 3).

Although clearly impactful, genic insertions of LINE-1 are arguably quite rare. How significant, then, is LINE-1 activity in cancer? The advent of massively parallel DNA sequencing has enabled researchers to examine the contribution of somatic retrotransposition in cancer at an unprecedented scale. A recent pan-cancer study performed whole-genome sequencing of 2954 cancer genomes across 38 histological subtypes [81]. These efforts revealed not only the widespread burden of LINE-1 retrotransposition in tumors, but that de novo insertions varied significantly across cancer types, further highlighting that TE activity is likely cell type-specific. One interpretation is that LINE-1 may simply be more expressed in retrotransposition-high cancers, such as epithelial tumors; alternatively, it is possible that retrotransposition-low cancers, like myeloid leukemias, are rather less tolerant of LINE-1 expression and/or insertional activity [82]. Multi-omic studies integrating LINE-1 expression analyses with de novo retrotransposition profiling across cancers will clarify the mechanistic basis for this heterogeneity. Nevertheless, in cancers with profound retrotransposition burden, de novo insertions were found to be frequent drivers of genomic structural rearrangements, some of which occasionally delete or amplify chromosomal regions carrying tumor suppressor genes or oncogenes, respectively [81]. Thus, LINE-1 ORF2p activity can contribute to cancer progression by promoting genomic instability and oncogenic structural variation in addition to mutagenesis of tumor suppressors (Fig. 3).

Beyond structural genetic changes, TEs can also contribute to cancer through epigenetic and *cis*-regulatory alterations. TE-encoded regulatory sequences, which are normally made inaccessible by repressive chromatin and DNA methylation, gain accessibility in cancer through epigenetic dysregulation, resulting in their occasional cooption for oncogenic *cis*-regulatory activity—a process termed ‘onco-exaption'. Lamprecht et al. demonstrated one of the first cases supporting such a model in Hodgkin’s lymphoma where a THE1B LTR element of the ancient ‘Mammalian apparent LTR Retrotransposon’ (MaLR) family was found to serve as an alternative promoter initiating transcription of the pro-oncogenic tyrosine kinase *CSF1R*, which is not expressed in normal B cells [83]. Analogous findings were made by Wolff et al. in bladder cancer and by Cruickshanks et al. in breast and colon cancers, where a LINE-1 promoter within the *MET* oncogene was found to be hypomethylated in tumors relative to normal cells inducing an alternative transcript [84, 85]. More recently, Jang et al. performed a large-scale transcriptomic study across 15 cancer types to assess the prevalence of TE onco-exaption events in cancers and identified 129 cases of novel TE cryptic promoter activity implicating 106 oncogenes [86]. Besides promoters, TEs can also act as enhancers. Deniz et al. analyzed public epigenomic and transcriptomic datasets from primary acute myeloid leukemia (AML) samples and cell lines and found that 6 ERV families have demonstrable genome-wide enhancer signatures, marked by DNase I hypersensitivity (a measure of open chromatin) and histone 3 lysine 27 acetylation (H3K27ac), in AML cells but not in normal blood lineages [87]. Importantly, CRISPR-based perturbation of a subset of these ERV loci validated their function as enhancers linked to the expression of known oncogenes [87].

To summarize, growing evidence support the model that TE activity is a unique feature of malignancy and can have profound impact on cancer genomes, both causal and contributory: (1) Retrotransposition activity can disrupt genes and/or structurally alter chromosomes to confer oncogenic potential; and (2) TE sequences can promote cancer progression without affecting the primary DNA sequence through acting as cryptic *cis*-regulatory elements such as promoters and enhancers. The outstanding challenge in the genomics era is to elucidate the pathways and principles governing how and why certain TEs but not others become reactivated and/or coopted in cancers and how cell type- and context-dependent cues influence this biology. Moreover, systematic functional testing at the individual locus level, such as the approach taken by Deniz et al., will be essential to validate any *cis*-regulatory contributions of TE sequences nominated by large-scale sequencing efforts. Lastly, although not discussed in this review, there is also notable evidence that TE proteins can have oncogenic function [88, 89]. Even more complex, non-coding functions of TE RNAs have also been identified in various biological contexts [50, 90–92], yet their implications in cancer have yet to be elucidated. Thus, it will be important for future studies to clearly define the relative oncogenic contributions of TE activity borne from their DNAs, RNAs, and/or proteins and whether their actions mainly interface with chromatin regulation or cytosolic pathways.

### Cancer-suppressive roles of TEs

While the biological consequence of TE activity has mostly been associated with transposition, a growing body of work suggests that this may not be the full picture. The process of retrotransposition generates nucleic acid intermediates that can trigger ancient cytosolic sensors evolved to detect invading viral genomes [93], which include the DNA-sensing cyclic AMP-GMP synthase (cGAS) and the RNA-sensing Retinoic acid Inducible Gene-I (RIG-I)-like receptors (RLRs) (Fig. 3). The precise molecular nature of the TE substrates engaged with these sensors remain incompletely understood but include double stranded RNAs (dsRNAs) generated by transcription of inverted Alu elements [94], bidirectional transcription of ERVs [95] and possibly LINE-1s [96], as well as complementary DNA (cDNA) derived from cytosolic reverse transcription of LINE-1 RNAs [97–99] by an unclear mechanism [100, 101].

The activation of the cGAS or RLR sensors triggers a type I interferon signaling cascade culminating in the induction of pro-inflammatory, anti-proliferative, and pro-apoptotic gene programs to mitigate apparent infection [102, 103]. Thus, retrotransposition in cancer presents a paradox: while insertional mutagenesis can promote cancer, the intermediates required to achieve transposition can have cancer-suppressing properties. The prevailing hypothesis to explain this apparent contradiction posits that cancer cells possess a tolerance threshold for TE expression and exceeding this threshold results in various toxicities including innate immune activation [93]; however, direct evidence for such a model is still lacking. If such a threshold exists, different cancer types may have varying tolerance for TEs. For example, myeloid leukemias are particularly susceptible to type I interferons. Cuellar et al. identified that AML cells silence retrotransposons via the H3K9 histone methyltransferase SETDB1 to mitigate interferon induction by TE-associated dsRNAs [104]. Conversely, cancers of epithelial origin appear relatively more tolerant of TE expression. Indeed, we have already discussed that epithelial tumors tend to acquire more de novo LINE-1 insertions. How, then, do these cancers upregulate TE activity without inducing innate immunity? One possibility is that cancers with high TE expression select for compensatory inactivating mutations in components of the type I interferon signaling cascade (Fig. 3). Consistent with this hypothesis, Zhao et al. used RNA-sequencing data from the Cancer Cell Line Encyclopedia (CCLE) to correlate the expression levels of TEs in lung cancers with mutations in factors involved in type I interferon signaling and identified a significant direct association [105], with the caveat that functional impact cannot be causally inferred from mutation burden alone.

Another possible explanation is that TE dsRNAs are modified by the adenosine-to-inosine RNA editor ADAR1 such that they no longer engage RLRs. Indeed, mutations in ADAR1 are a known genetic cause of TE dsRNA-dependent type I interferons which drive autoimmune diseases like Aicardi-Goutières syndrome and systemic lupus erythematosus [98, 106]. Conversely, there is also evidence that ADAR1 overexpression correlates with cancer progression, although the precise mechanisms are unclear [107]. Nevertheless, the findings by Zhao et al. and others [93, 108, 109] contribute to an emerging model in which TEs represent coopted genomic “sentinels” that sense epigenetic dysregulation within pre-neoplastic cells and trigger their demise through interferon activation; thus, pre-malignant cells must overcome this innate barrier imposed by TEs to transform into frank neoplasia. Paradoxically, this same mechanism ablating pre-neoplasia has been shown to promote so-called “sterile inflammation” in mouse models of aging [97, 99], wherein the progressive erosion of heterochromatin with age results in de-repression of TEs and aberrant interferon activation, exacerbating aging-associated pathologies and cellular senescence across numerous tissues. Thus, adding to their multi-faceted functions in cells, TEs also act as potent immune modulators that normally safeguard against tumorigenesis, but, when gone awry with age, inadvertently accelerates pathology.

Besides modulating innate immunity, TE activity can also impact cancer-initiating cell activity through interfacing with the DNA damage response pathways in specific cancer types including myeloid leukemias [82] (Fig. 3). Maintenance of genomic stability is required for the self-renewing capacity of cancer stem cells such as AML-initiating cells [110]. Loss of genome integrity caused by inactivation of DNA damage response proteins (i.e. ATM and BRCA1) [111] or certain epigenetic regulators (i.e., MLL4 and LSD1/KDM1A) [111–115] promotes differentiation of AML-initiating cells. LINE-1 retrotransposition can induce genomic instability by creating single- or double-strand DNA breaks [116, 117], which activates DNA damage response pathways culminating in cell cycle exit and apoptosis. As such, myeloid leukemias were found to have enhanced suppression of LINE-1s at least in part through epigenetic silencing mediated by the Human Silencing Hub (HUSH) complex, whereas reactivation of evolutionarily young LINE-1s selectively impairs the propagation of myeloid leukemia-initiating cells [82].

### Modulating TE activity for anti-cancer therapy

There is now substantial interest in manipulating TE activity for cancer treatment [93, 118]. Pharmacologic approaches to modulate TE expression rely on so-called “epigenetic drugs”, such as DNA methyltransferase inhibitors (DNMTi) or hypomethylating agents (HMAs). However, the TE-centric rationale for the usage of DNMTi is a relatively recent concept. Compounds that are recognized today as DNMTi, such as 5-azacytidine (5-aza), have existed since the early 1960s, originally intended as general chemotherapies with unclear mechanisms of action [93, 119]. Initially rejected by the FDA as a cytostatic drug in the 1970s due to significant toxicities at high dosages, 5-aza was later shown in pioneering work by Jones and Taylor to demethylate DNA when used at low doses for longer durations [120]. Their initial studies and subsequent validation work led to the FDA approval in 2004 of low-dose 5-aza for the treatment of the myelodysplastic syndromes (MDS) [119]. Since then, DNMTi have proven to be especially efficacious drugs for myeloid malignancies and are now part of the standard of care guidelines for MDS. Though their mechanism of action has long been nebulous, DNMTi have been presumed to work by reactivating hypomethylated tumor suppressor genes. [121]. However, in 2015, two studies by Roulois et al. [122] and Chiappinelli et al. [95] provided evidence that low-dose DNMTi treatment demethylates TE loci, specifically ERVs, producing dsRNAs that induce an anti-proliferative type I interferon response, a mechanism termed “viral mimicry” [93] (Fig. 3). Subsequent studies have established correlations between reactivation of different TE families and the viral mimicry response in various cancer contexts [96, 104, 123, 124]. Yet, crucially, most findings to date remain largely associative without causal verification that a given reactivated TE species in fact ligates a nucleic acid sensor(s). Of note, a recent study by Medhipour et al. profiled MDA5-associated RNAs upon treatment of patient-derived colorectal cancer cells with epigenetic inhibitors and identified inverted-repeat Alus as the dominant drug-induced immunogenic dsRNA ligand [94]. The identification of inverted Alus as the key mediator substrate allowed the authors to hypothesize an involvement of ADAR1, as inverted Alus are known ADAR1 substrates, and, indeed, they uncovered that ADAR1 co-inhibition synergized with epigenetic therapy to augment the viral mimicry response. Notably, transcriptomic analyses also identified upregulation of ERVs upon epigenetic therapy treatment yet with minimal engagement of MDA5, highlighting the need for functional testing to parse out changes in TE expression that are directly immunogenic from those that are not.

This type I interferon response induced by viral mimicry has pleiotropic consequences on cancer cells. In addition to activating anti-proliferative and pro-apoptotic pathways, type I interferon signaling also concomitantly upregulates MHC class I antigen presentation machinery, which are normally expressed in all somatic cells but often become silenced in tumors for immune evasion (Fig. 3), suggesting viral mimicry may augment immunotherapy response [125, 126]. Indeed, recent studies support the notion that concurrent upregulation of TEs and antigen presentation genes upon viral mimicry induction results in increased presentation of TE-derived peptides on tumor cells, providing a novel source of tumor-associated “neoantigens” to signal cellular immunity [127, 128]. Griffin et al. performed an in vivo CRISPR screen to discover epigenetic modulators of immune checkpoint blockade (ICB) response and identified the repressive histone methyltransferase SETDB1 as a potent mediator of ICB resistance in mouse melanoma and lung carcinoma models [129]. Knockout of *Setdb1* in mice resensitized tumors to ICB treatment in a CD8 + cytotoxic T-cell dependent manner. Subsequent MHC I immunopeptidomics and TCR repertoire analysis of tumor infiltrating T lymphocytes identified numerous TE-encoded antigenic peptides loaded onto MHC I on the surface of tumor cells as well as expansion of TCR clonal diversity, presumably recognizing TE-derived peptides; however, definitive evidence of TE antigenic binding by putative TCRs was not demonstrated. In another study, Zhang et al. demonstrated SETDB1 as a critical mediator of immune evasion in an independent mouse melanoma model and, furthermore, identified the histone H3 lysine 4 demethylase KDM5B as the chromatin factor that recruits SETDB1 to silence TEs in a demethylase-independent manner [130]. Interestingly, both groups detected ERVs from the MMVL30 family among the top upregulated TEs upon *Setdb1* or *Kdm5b* knockout, suggesting that certain TEs may be more susceptible to epigenetic perturbations and/or have higher antigenic presentation potential.

Other aspects of the TE replication cycle are also emerging as targetable vulnerabilities and potentially synergize with epigenetic therapies. Rajurkar et al. recently showed that treatment of colon cancers with nucleoside reverse transcriptase inhibitors (NRTI), normally used as antiviral agents, reduced tumorsphere formation in vitro [131]. RNA sequencing analyses showed that NRTI treatment induced type I interferon signaling and DNA damage response pathways. Surprisingly, cells treated with NRTI had reduced cytosolic cDNAs but instead accumulated RNA:DNA hybrids; importantly, the authors demonstrated that although inhibition of TE-dependent reverse transcription reduces canonically immunogenic cDNAs, the RNA:DNA hybrids that accumulate retain immunogenicity in a STING-dependent manner [131]. NRTI treatment also increased replication stress possibly by producing retrotransposition intermediates that cannot be efficiently repaired, triggering the observed DNA damage response. The dual induction of interferon and DNA damage signaling by NRTIs suggested these cells would be especially susceptible to combined therapy with 5-aza and/or DNA damage-inducing chemotherapies. Indeed, the authors tested combinations of NRTIs with 5-aza or 5-fluorouracil/oxaliplatin and showed synergistic cytotoxicity in vitro. Importantly, all the anti-cancer effects observed were dependent on mutant p53 status, highlighting the influence of genotype in determining the consequences of TE activity in cancer. This notion is further emphasized by work from Ardeljan et al. wherein they found that LINE-1 overexpression is incompatible with cellular growth of non-transformed cells harboring wildtype p53 partly due to retrotransposition-mediated replication stress, suggesting LINE-1 upregulation may represent an early selection pressure by which some cancers acquire p53 mutations [132, 133]. Interestingly, myeloid leukemias seem to behave oppositely to epithelial cancers, in that their sensitivity to LINE-1 expression is dependent on p53; moreover, treatment of AML cell lines with the NRTI lamivudine blocked LINE-1 overexpression-mediated effects [82]. Thus, the therapeutic manipulation of TE activity will certainly need to be tailored with genotype- and cancer type-specific consideration.

In summary, emergent TE-centric therapeutic strategies hold significant promise as potential single-agent and/or combination cancer treatments. Multiple aspects of the retrotransposon life cycle are targetable: (1) epigenetic compounds are effective at derepressing repeat-derived nucleic acids to induce viral mimicry in many tumor types; (2) reverse transcriptase (RT) inhibition with nucleoside analogs such as NRTIs induces accumulation of immunogenic RNA:DNA hybrids at least in p53-mutated colon cancers; and (3) RT inhibition can also induce replication stress and activation of the DNA damage response in epithelial cancer cells. Future translational efforts will be needed to determine the ideal combinations of targets to pharmacologically manipulate TE activity for cancer treatment. Mechanistically, more studies are needed to define the molecular basis of the cancer type-specific responses to TE activity, such as the opposing effects to RT inhibition by epithelial tumors versus myeloid leukemias. More work is also needed to clarify which molecular sensors sense TE nucleic acids, as mechanisms for both DNA and RNA sensing have been proposed, as well as which TE families (and specific genomic loci) produce direct immunogenic substrates, perhaps operating in a cancer type-dependent manner.

## Advances in experimental and computational methods to study TEs

**Fig. 4:**
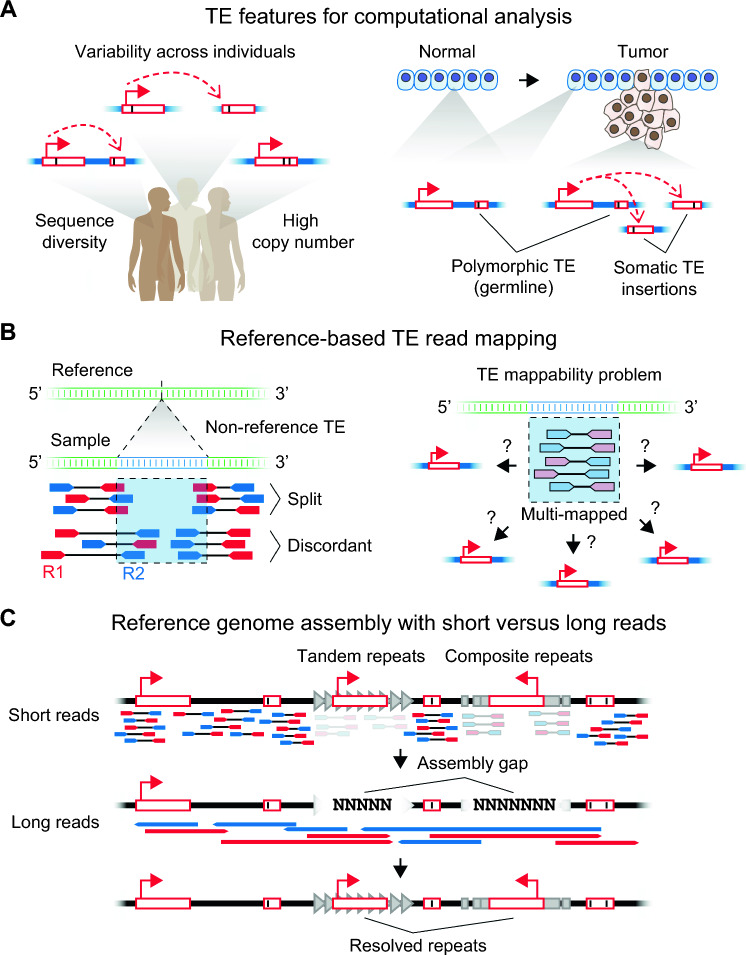
Computational analysis of TE genomic variation and expression using short-read and long-read sequencing.** A** TE analysis is challenging because of their high copy number, sequence diversity, and variability across individuals**.** These problems are exacerbated in cancer with increased polymorphic TE content and structural variation; moreover, somatic TE inserts in the tumor must be distinguished from germline variants. Internal black lines depict nucleotide variants within TEs. **B** Reference-centric approaches for detecting putative de novo TE insertions based on alignment characteristics of reads spanning the TE insert (split versus discordant reads). The vertical dashed line depicts the breakpoint of an inserted TE (blue). Sequencing reads aligning entirely within TEs often match identically with multiple genomic copies, resulting in poor mappability. R1, read 1. R2, read 2. **C** Long-read sequencing has improved reference genome assembly, bridging gaps (“NNN”) in reference genomes assembled by short-read technologies. These gaps typically are composed of complex repetitive elements such as tandem repeats or multiple nested TEs (composite)

TEs are challenging to study given their high copy numbers, diverse sequence forms, and variability across individuals (Fig. 4A). The advent of massively parallel sequencing or so-called “next generation sequencing” (NGS) has made the task of studying TEs ever more tractable, yet still demands significant expertise [134, 135]. These problems are amplified in cancer genomes, which frequently undergo radical chromosomal alterations and accumulate significant structural variations during tumorigenesis, in part derived from TE activity. NGS approaches are limited by short sequencing read lengths, precluding the full resolution of repetitive DNA. Thus, the ability to accurately sequence long nucleic acid molecules is critical to fully dissect the functions of TEs and other repetitive elements in cancer genomes. It is important to note the major differences in the sequencing chemistries underlying NGS and emerging long-read sequencing platforms: NGS is based on a “sequencing-by-synthesis” (SBS) chemistry in which nucleic acid molecules of interest are sequenced via a base-by-base incorporation-detection-cleavage reaction. Conversely, long-read sequencing employs a different sequencing chemistry based on either 1) in the case of Pacific Biosciences (PacBio)-based “SMRT” systems, the real-time detection of light emitted upon polymerase incorporation of labeled nucleotides on a single nucleic acid molecule within microscopic wells, or 2) in the case of Oxford Nanopore Technologies (ONT), the detection of characteristic electrical current alterations, which correspond to specific nitrogenous bases, as a nucleic acid molecule traverses a “nanopore” protein embedded within a conductive surface. Importantly, these chemistries enable the direct sequencing of long native DNA or RNA molecules including modified bases. Initially plagued by high basecalling error rates [136], long-read sequencing technologies have seen rapid improvements in accuracy in the past few years and are reaching an inflection point towards widespread adoption [137]. Already, long read-based genomics methodologies are being devised to study the contributions of TEs in human biology and non-human model organisms [138]. In this section, we describe the specific experimental and computational challenges involved in studying TEs in cancer with respect to three themes: structural variation, expression analyses, and epigenetic modifications. We discuss the conceptual basis by which current NGS-based tools approach these problems and illustrate how emerging long read-based assays are overcoming many limitations of existing methods.

### Identifying de novo TE-mediated structural variation in cancer genomes

TE-mediated variants in cancer genomes can range from the simple scenario of single de novo insertions to more complex cases where recombination of homologous repeats generate large-scale chromosomal rearrangements. Most cancer genomics studies rely on reference-centric approaches to map sequencing reads with the assumption that most of the genomic DNA of the sample of interest generally matches the genomic reference. While effective for many applications, this approach is problematic for repetitive DNA because sequencing reads derived from repeats are, by definition, highly similar in nature, resulting in ambiguous alignments. This is particularly problematic for the youngest TE subfamilies, such as the human-specific LINE-1 subfamily (L1Hs), which are often nearly identical in sequence and present at high copy numbers throughout the genome. Because the youngest subfamilies are also the only active TE loci, polymorphic germline insertions are present across individuals and de novo insertions in tumors, yet both would be absent from the reference genome; these insertion types are termed “non-reference” (Fig. 4B). Moreover, the commonly used GRCh38 human reference genome still has significant gaps in its consensus sequence, primarily in the highly repetitive telomeric and pericentromeric regions composed of nested arrays of diverse repeats [139]; thus, otherwise active TEs located in these regions would remain unknown (Fig. 4C). All these factors together significantly hinder our ability to identify TE-mediated structural variations in cancer genomes.

**Fig. 5:**
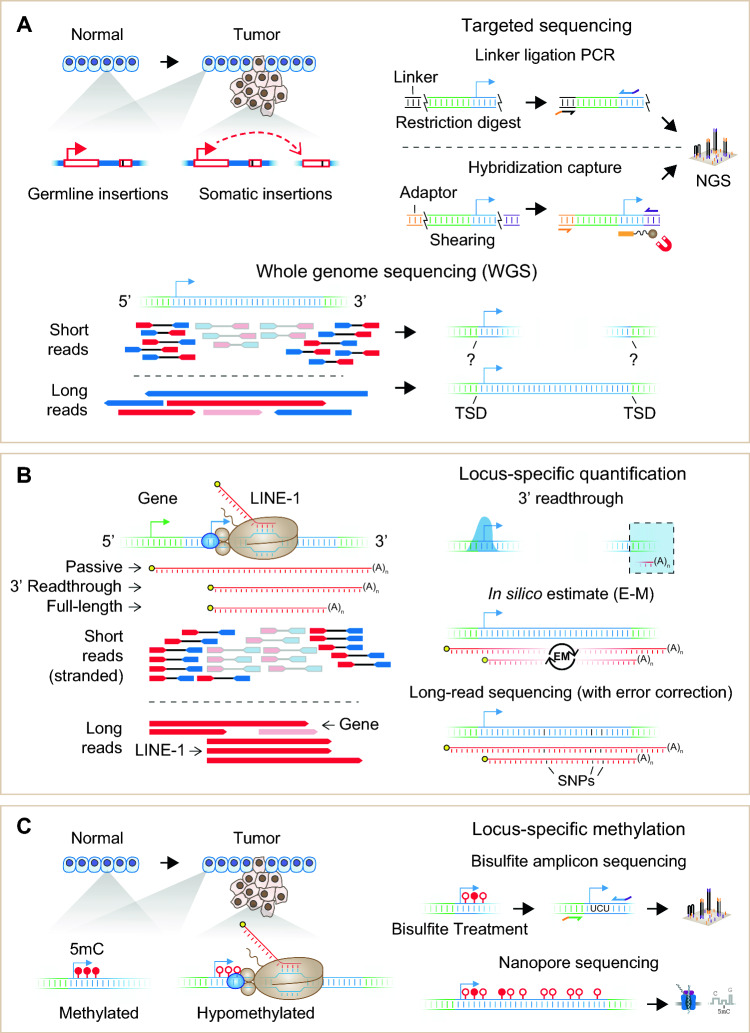
Experimental strategies to detect TE variation, expression, and epigenetics. **A** Somatic TE insertions in cancer can be detected by whole genome sequencing (WGS) or targeted approaches that enrich for TE sequences (linker ligation PCR versus hybridization capture). NGS, next-generation sequencing. TSD, target site duplication. **B** TE expression analysis is complicated by multiple potential sources of TE-containing RNAs, particularly for intronic TEs. Specific TE loci often cannot be distinguished with short reads unless containing sufficient unique sequence content (3′ readthrough method). In silico methods can estimate locus-specific TE expression by rescuing multi-mapped reads. With sufficient accuracy, full-length TE long reads can distinguish individual TE loci by virtue of characteristic SNPs as well as identify TE-initiated transcripts versus passive readthrough by the host gene using 5′ end transcription start site (TSS, colored arrowheads) information. E–M, expectation–maximization algorithm. **C** Cancer genomes frequently undergo DNA hypomethylation during tumorigenesis, hence DNA methylation is commonly measured to assess the epigenetic permissivity of TEs in cancer. Locus-specific methylation can be detected using bisulfite conversion of genomic DNA paired with locus-specific amplicon sequencing. Nanopore sequencing enables direct detection of modified nucleotides, including 5-methylcytosine (5mC), during basecalling

Whole genome sequencing (WGS) with paired-end reads is the most common approach for identifying cancer-associated TE variants (Fig. 5A). Due to the short read lengths (typically 50–150 base pairs on each end), NGS-based WGS approaches require significant computing power and specialized computational tools to detect TE variants by analyzing the alignment patterns of paired-end reads relative to the reference genome. There are two general strategies to identify TE-mediated insertions or rearrangements: the “split read” and the “discordant pair” approach. The “split read” approach looks for alignment gaps in reads on either ends relative to the reference (Fig. 4B). In other words, the TE containing read(s) would partially align with the unique genomic segment adjacent to the TE as well as a contiguous but ambiguously mapped portion coming from the repeat itself. The “discordant pair” approach focuses on identifying read pairs in which one read end aligns uniquely whereas the other read is entirely ambiguous corresponding to a repeat alignment. In practice, both strategies are often used in concert by specialized software to identify putative TE variants with high confidence. Two popular tools to parse paired-end reads for cancer-associated TE insertions are MELT [140], which detects insertion polymorphisms of TEs in both somatic and germline samples, and TraFiC-mem [141], which specializes in identifying somatic insertions using pairwise comparisons of control versus tumor samples. Numerous tools have been introduced for genotyping specific types of TE insertions (Table 1), leading to the identification of thousands of polymorphic TE insertions in different human cancer datasets. While efforts have been made to benchmark these software [142, 143], upfront effort is still warranted to determine the most suitable tool(s) to employ depending on the specific characteristics of the sequencing data and specific hypothesis being investigated.

**Table 1:** Computational methods to study TE genomic variation, expression, and epigenetics

Methods	Main applications	Input files	PMID or DOI
*TE structural variation*			
Tea	Detects de novo TE insertions by comparing tumor/normal samples	BAM alignment files	22745252
RetroSeq	Detects de novo TE insertions in samples, low computer memory requirement	Paired-end BAM	23233656
Alu-detect	Detects de novo insertions of Alu elements	Paired-end FASTQ, SAM or BAM	23921633
TraFiC	Detects de novo retrotransposition and 3′ transductions in cancer genomes from matched tumor/normal samples	Paired-end FASTQ and reference genome FASTA	25082706
TranspoSeq	Detects de novo retrotransposon insertions in cancer genomes	BAM for each tumor and normal sample	24823667
Mobster	Detects de novo TE insertions by clustering clipped and/or discordant mapped reads	Single-end or paired-end BAM	25348035
Jitterbug	Detects de novo TE insertions in matched tumor/normal samples	BAM and TE annotation GFF file	26459856
MELT	Detects polymorphic TE insertions at population scale	BAM and custom config file	28855259
TIF_finder	Fast detection of TEs in cancer genomes	Paired-end FASTQ and reference genome FASTA	32917036
PALMER	Detects non-reference mobile element insertions (LINE, Alu, SVA, HERVK) with a pre-masking approach; able to detect TSDs, 5′ inversions, 3′ transduction, and polyA tails	BAM, indexed FASTA of reference genome	31853540
xTea	Detects both germline and somatic TE insertions from multiple NGS platforms with ability to annotate complex SV types	List of BAM and GFF	34158502
nanomonsv	Detects somatic SVs, including complex mobile element insertions, using long-read sequencing from matched tumor/normal samples	Minimap2 BAM, reference genome FASTA	37336583
Somrit	Detects somatic TE insertions from long reads using a reference-based local realignment procedure	Minimap2 BAM, long-read FASTQ, reference genome FASTA	https://doi.org/10.1101/2023.08.06.552193
GraffiTE	Detects polymorphic TEs in genome assemblies or long read datasets using a pangenomic approach (under beta testing)	Minimap2 BAM, reference genome or assembly FASTA	https://doi.org/10.1101/2023.09.11.557209
*TE expression*			
RepEnrich	Quantifies expression of repeats including LTRs	BAM from bowtie and custom BED file of repeats annotation	25012247
TEtranscripts	Most popular tool, uses expectation–maximization algorithm, performs differential expression analysis of TEs and genes, TE sub-family, and locus level expression	BAM files from STAR and TE annotation GTF files	26206304
TEtools	TE differential expression from unannotated and unassembled genomes	Paired-end FASTQ, TE sequences in FASTA and rosette (two columns annotation) file	28204592
SalmonTE	Computes TE expression and reports as transcripts per million reads (TPM)	Single-end or paired-end FASTQ and CSV with sample info	29218879
ERVmap	Specialized tool for ERV expression analysis	Single-end or paired-end FASTQ	30455304
SQuIRE	Locus-specific and family-scale TE expression based on expectation–maximization algorithm	Paired-end FASTQ, repeat annotations (TE_ID, BED or GFF)	30624635
*REdiscoverTE*	Comprehensive expression analysis of all repeats including TEs	“REdiscoverTE.tsv” file from processing of RNA-seq alignments and reference genome	31745090
LIONS	TE-initiated transcripts in group-wise comparisons	Paired-end FASTQ	30793157
TeXP	Transcriptional activity of LINE-1 elements in different cell lines	Single-end FASTQ	31425522
Telescope	Locus-specific analysis of TE expression, reassigns ambiguous mapped reads using a statistical model	SAM and TE annotation GTF	31568525
L1EM	Locus-specific analysis of L1Hs expression based on expectation–maximization algorithm	Paired-end BAM, BWA indexed reference genome FASTA	31584629
SoloTE	Locus-specific analysis of TE expression from scRNA-seq data	BAM, TE annotation in BED	36202992
Unnamed Method	Transcript assembly strategy to improve TE quantification from scRNA-seq data	Paired-end FASTQ from bulk RNA-seq and scRNA-seq data	33355230
LocusMasterTE	Uses improved mappability of long-read RNA-seq data as a prior for expectation–maximization reassignment of short-read RNA-seq data	SAM or BAM from Telescope, GTF of TE loci, TPM gene count from long read RNA-seq data	https://doi.org/10.1101/2023.03.21.533716
*Epigenome and 3D genome*			
TLDR	Detects DNA methylation from ONT sequencing data	Minimap2 BAM, reference genome FASTA; FAST5 raw data for methylation calling	33186547
PAtChER	Locus-specific protein enrichment profiles at TEs and other repeats by integrating ChIP-seq and HiChIP data	Paired-end FASTQ of HiChIP data and reference genome FASTA	34908129
scTE	TE expression and chromatin accessibility analyses from scRNA-seq and scATAC-seq data	BAM, TE annotation in BED, GTF of genes	33674594
T3E	Family and sub-family level epigenetic profiling of TEs between samples	BAM	36451223
TEpeaks	A package from TEtranscripts for narrow ChIP-seq peak-calling from both uniquely mapped and multi-mapped reads	BAM	26206304
mHiC	Reassigns multi-mapped reads from Hi-C data to improve coverage at repetitive regions	Paired-end FASTQ, reference genome FASTA	30702424
HiTea	Detects non-reference insertions of the major human retrotransposons, Alu, L1Hs, and SVA using Hi-C data	PAIRSAM or BAM, TE-family consensus sequences FASTA, TE-family annotation in BED	33136153

Although effective, WGS requires large amounts of sequencing reads to have sufficient coverage to call TE variants, which are often present at lower allelic frequencies in a tumor sample. To address this issue, targeted sequencing approaches have been devised to enrich sequencing reads containing TE(s) of interest (Table 2). These techniques differ in their enrichment strategies, but broadly encompass three general strategies: 1) linker-ligation PCR, 2) oligonucleotide (oligo) hybridization capture, or 3) hybrid methods that combine aspects of the other two approaches (Fig. 5A). Notable methods based on linker ligation-PCR include the Amplification Typing of L1 Active Subfamilies (ATLAS) [144, 145] and Transposon Insertion Profiling (TIP)-seq [146]. These techniques follow the general principle of digesting genomic DNA using restriction enzyme(s) (RE) that cut frequently enough to produce short fragments containing the 5′ or 3′ ends of a TE contiguous with its unique genomic flank, followed by ligating short DNA adaptors with known sequences to the cut ends. A subsequent amplification step using PCR primers specific to the ligated adaptor and TE(s) of interest is employed to enrich for sequencing of inserts harboring TE(s) of interest. These TEs can include known reference inserts as well as de novo insertions. The major difference between ATLAS-seq and TIP-seq is how they approach selectively amplifying TE-containing fragments: ATLAS-seq employs a “suppression PCR” strategy while TIP-seq leverages a “vectorette” linker design. In suppression PCR, the linker sequence on each genomic fragment ends anneals to itself, forming a “panhandle” structure, only to be released upon forward primer extension. In contrast, vectorette linkers contain homology to the linker-specific reverse primer on the reverse strand, thus only allowing primer annealing upon forward primer extension. Nevertheless, a limitation of either approach is that it is often difficult to predict the frequency of RE cutting sites at non-reference loci, therefore loci that cannot produce short enough insert sizes to be compatible with PCR and/or NGS are missed.

**Table 2:** Experimental methods to study TE genomic variation, expression, and epigenetics

Methods	Main applications	TE types	PMID or DOI
*TE structural variation*			
L1-seq	Detection of de novo retrotransposition by linker-ligation PCR	L1Hs	26895047
ATLAS-seq	Detection of de novo retrotransposition by “suppression” PCR method	L1Hs	26895048
TIP-seq	Detection of de novo retrotransposition by “vectorette” PCR method	L1Hs	30899333
RC-seq	Detection of de novo retrotransposition by hybridization capture enrichment	L1Hs	26895046
SeqURE	Detection of de novo retrotransposition by hybridization capture and target-specific PCR	Alu and L1Hs	33317630
ME-Scan	Detection of de novo retrotransposition by hybridization capture and target-specific PCR	Alu, SVA, LINE-1	32110248
REBELseq	Detection of de novo retrotransposition by linker-ligation PCR	L1Hs	32132168
Cas9 targeted enrichment of mobile element insertions	Cas9-assisted target enrichment of de novo mobile element insertions for ONT sequencing	Alu, SVA, LINE-1	34117247
NECO-seq	Detection of de novo retrotransposition by linker-ligation PCR with single-neuron nuclei enrichment and whole genome amplification	L1Hs	36173571
*TE expression*			
LINE-1 3′ readthrough	Locus-specific expression of L1Hs by measuring 3′ readthrough	L1Hs	27016617
SCIFER	Single-cell profiling of LINE-1 expression by short-read sequencing, based on 10X 3′-cDNA sequencing	L1Hs	36028901
CELLO-seq	Profiling the expression of full-length TEs using single-cell long-read sequencing	All TEs	34782740
capTEs	Cas9-assisted quantification of expression patterns of locus-specific TE transcripts	All TEs	37741908
scL1-seq	Single-cell profiling of LINE-1 expression by short-read sequencing, based on 10X 5′-cDNA sequencing	L1Hs	36744437
*Epigenome and 3D genome*			
Locus-specific LINE-1 DNA methylation profiling	Evaluation of methylation levels of individual L1Hs promoters by bisulfite conversion and amplicon sequencing	L1Hs	31230816
bs-ATLAS-seq	Evaluation of methylation levels of L1Hs promoters by bisulfite conversion and ATLAS-seq	L1Hs	36449162
dCas13 targeted m6A demethylation	Targeted m6A demethylation of TE RNAs	TEs	34108665, 35511947
Nanopore-DamID	Simultaneous profiling of DNA methylation and TF occupancy by ONT sequencing	TEs	https://doi.org/10.1101/2021.08.09.455753
NanoNOMe-seq	Simultaneous profiling of DNA methylation and chromatin accessibility by ONT sequencing	All TEs	33230324
scTEM-seq	Targeted analysis of TE methylation levels at single-cells level	All TEs	35388081
HiChIP and PAtChER	Combination of HiC and ChIP-seq for locus-specific chromatin profiling of interspersed repeat loci	All TEs	34908129
4Tran	Adaptation of 4C-seq and Capture-3C assays for profiling of long-range chromatin interactions at specific TE loci	ERVs, all TEs	30541598
*Functional perturbation*			
CRISPR-Cas9 editing	Genome editing for functional analysis of TEs in mammalian cell lines	All TEs	36449171
CRISPRi	Epigenomic editing to inhibit locus-specific gene expression by CRISPR-mediated transcriptional repression	LINE-1 (human, mouse), HERV	32665538, 37308596, 36610399
CRISPRa	Epigenomic editing to activate locus-specific gene expression by CRISPR-mediated transcriptional activation	LINE-1 (human, mouse), HERV	33833453, 36070749, 37591949, 36610399
TALE-based epigenetic modification	Altering expression level of TEs in mammalian cells	LINE-1 and satellite repeats	36449170

Hybridization capture is an alternative to linker-ligation PCR that, in place of RE digestion, uses physical shearing (*e.g.*, sonication) to fragment DNA to NGS-compatible sizes followed by sequencing adaptor ligation and nucleic acid hybridization using TE sequence-specific oligos (Fig. 5A). Retrotransposon Capture-seq [147, 148] uses single-stranded DNA probes specific to the 5′ and 3′ ends of the L1Hs consensus sequence to enrich for L1Hs-containing genomic DNA fragments. The covalent linkage of a biotin moiety to the hybridization probes during its synthesis allows for streptavidin capture of hybridized fragments followed by stringent washing to deplete non-target DNA fragments. This approach is particularly beneficial for lower-input material such as primary tissues, because samples can be processed with sample-specific barcodes within the ligated linkers during pre-capture library preparation followed by pooling of multiple samples during hybridization capture steps. It is important to note that no method is perfect; both linker ligation-PCR and hybridization capture approaches are susceptible to potential artifacts during sample processing resulting in false positive insertion calls [149]. Thus, non-reference insertions should be properly validated by genotyping PCR and Sanger sequencing with primers flanking the putative insertion site.

Long-read sequencing is transforming our ability to sequence and align repetitive DNA, significantly simplifying the task of identifying structural variants [138, 139, 150, 151]. Long-read platforms can produce on average read lengths of 10–25 kilobases using PacBio systems and 10–100 kilobases on ONT-based sequencers [139], easily spanning the longest TEs in the human genome including full-length LINE-1 elements (Fig. 5A). Importantly, what was once the biggest trade-off of long reads technologies, accuracy, is no longer limiting. The latest PacBio “HiFi” chemistry produces reads with average Q30 accuracy scores, meaning basecalling errors occur once every 1000 bases (*i.e.*, 99.9% accuracy), whereas ONT’s R10.4.1 pore chemistry can routinely achieve Q20 scores or 1 error in 100 basecalls (*i.e.*, 99% accuracy). The major limitation of current long-read platforms, however, remains its modest throughput of tens of millions of reads per run compared to NGS. Still, useful workarounds have been devised, such as the use of CRISPR/Cas9 for targeted sequencing of regions of interests by cutting and ligating sequencing adaptors in vitro only onto fragments targeted by CRISPR guide RNAs; this strategy was demonstrated recently to improve nanopore sequencing coverage at TEs and, importantly, was able to detect non-reference TE insertions [152]. Another powerful demonstration of the promise of long-read sequencing is the recent completion of the telomere-to-telomere (T2T) human reference genome, which has filled in the remaining missing sequences of the GRCh38 reference to achieve the first complete representative assembly of human genomic DNA [153]. Importantly, this milestone was only possible with the adoption of long reads to span the complex, highly repetitive DNA regions that were virtually impossible to scaffold using NGS technologies alone (Fig. 4C). The T2T reference has revealed previously unknown repeats including 13 interstitial satellite arrays and 19 composite elements (repeat unit consisting of three or more types of repeats) [153].

Software development for detecting TEs from long reads is still in its infancy (Table 1). A recently developed tool 'nanomonsv' has been tailored for the precise identification of complex mobile element insertions in cancer versus non-cancerous samples with single-base resolution [154]. In another study, Pascarella et al. developed an analysis pipeline called “TE-reX” to detect recombination events involving Alu and LINE-1 sequences with support for both long reads and hybridization capture NGS reads [155]. Currently, PALMER [156], GraffiTE [157] and xTea [158] represent a few dedicated tools developed for the detection of TE insertions using long-read sequencing data. PALMER leverages PacBio HiFi reads to detect L1Hs insertions, improving the detection of de novo LINE-1 insertions, especially those nested within complex repetitive genomic regions; specifically, PALMER leverages a targeted pre-masking approach, an annotation strategy that “masks” repetitive regions by transforming their sequences into ‘N’ nucleotides to reduce alignment complexity [156]. The xTea software offers a new approach for identifying TE insertions from long reads, with the ability to identify full-length polymorphic copies of LINE-1 within highly complex regions such as centromeres [158]. xTea detects TEs insertions from multiple sequencing platforms, using discordant or clipped reads from short reads, local assembly of each candidate TE insertion site from long reads, and/or grouping the TE associated sequences according to their molecular barcodes from 10X Genomics linked reads as input. Lastly, GraffiTE is a software package currently under beta testing which can genotype different types of TE variants with high precision from both short- and long-read data by using a pangenomic “graph”-based approach [157, 159].

### Quantifying TE expression

Measuring genomic locus-specific expression of TEs remains a formidable challenge with current NGS-based RNA sequencing (RNA-seq) [134]. The challenges are three-fold: First, TEs are often embedded within introns of host genes, resulting in uncertainty as to whether RNA reads mapping to an intronic TE truly stems from the TE itself or is a byproduct of the transcribing host gene, so-called “passive readthrough” (Fig. 5B). Second, despite being upregulated in cancer, TEs are expressed at relatively low levels compared to host genes resulting in potential detection and dropout issues depending on sequencing depth. Third, whereas TE DNA reads can contain adjacent unique genomic DNA to anchor the repetitive DNA, TE RNA reads derive mostly from within the repeat transcription unit, significantly reducing mappability rates. These factors influence the resolution and accuracy with which expression information can be extracted from TE RNA reads. In recent years, the first and second challenges have mostly been addressed by the standardization of stranded library preparation workflows along with deeper sequencing. The addition of strand information allows for more precise assignment of RNA reads overlapping with intronic TE loci oriented antisense to its host gene; however, RNA reads mapping to TEs in sense with their host gene are still indistinguishable.

Mappability, on the other hand, remains a major limitation of current NGS-based approaches for TE expression analysis (Fig. 5B). TE RNA reads are often “multi-mapping” in that they align to multiple loci in the reference genome (Fig. 4B). Because most studies prioritize high-confidence alignments, ambiguous or multi-mapping reads are typically discarded, resulting in loss or biasing of information on TE expression [134]. This is particularly problematic for studying the youngest TE subfamilies, which tend to be the most upregulated in cancer, since these loci are nearly identical and thus produce mostly multi-mapping alignments. Nevertheless, most sequence aligners have optional parameters to handle or “rescue” multi-mapping reads. The options vary slightly between aligners but generally allow for discarding multi-mapped reads entirely (i.e., retain only uniquely mapped reads), randomly picking one multi-mapped alignment per read, or keeping all multi-mapped alignments per read. Several studies have benchmarked existing RNA-seq aligners to identify optimal parameters for accurate quantification of TE transcripts (Table 1). For example, Teissandier et al. performed a head-to-head comparison of the most popular RNA-seq aligners (Bowtie [160], Bowtie2 [161], BWA aln [162], BWA mem [162], STAR [163], and Novoalign) using real and simulated datasets [164]. They found that keeping multi-mapping reads was essential for high accuracy of TE quantification and that, while all the tested aligners performed similarly in terms of accuracy when keeping multi-mapped reads, the STAR aligner outperformed the others in terms of memory usage and speed. Moreover, the mappability of paired-end reads were vastly superior to single-end libraries. These improvements have enabled subfamily level quantification of TE expression, where reads mapping to annotated TE loci of the same subfamily are counted in aggregate, allowing for differential expression analysis of TE levels between samples or conditions of interest.

Given that TE activity is known to derive from a subset of source genomic loci, measuring the expression of individual TE copies has been a long-sought goal. An early approach for locus-specific LINE-1 expression was proposed by Philippe et al. where they only count RNA-seq reads from the unique regions downstream of LINE-1 loci as a proxy for locus-specific expression, with the assumption that this 3′ transcriptional readthrough signal is proportional to the activity of the LINE-1 transcription unit [68]. These loci are further filtered by the presence of histone 3 lysine 4 trimethylation (H3K4me3) signal in the 5′UTR, which marks active promoters (Fig. 5B). Indeed, this rationale forms the basis of how the source elements of de novo LINE-1 retrotransposition events are identified as the 3′ genomic flank of donor elements is often transduced to daughter loci by virtue of 3′ readthrough transcription [141]. However, an important caveat of this approach is that the polyA signal varies in strength between different LINE-1 copies, thus, not all loci have detectable 3′ readthrough [165]. Alternatively, in silico strategies have been proposed to estimate locus-specific TE expression from NGS-based RNA-seq data (Table 1). TEtranscripts [166], SQuIRE [167], and L1EM [165] are commonly used software for TE quantification which operate based on expectation–maximization (E–M) algorithms used to statistically redistribute multi-mapping reads to their most likely alignment. These software generate largely concordant results [164], differing mostly in their implementation. SQuIRE is an end-to-end workflow that requires inputs of raw FASTQ sequencing files and uses STAR for read mapping. L1EM and TEtranscripts allow user flexibility for aligner choice, requiring only BAM alignment files as input. All three software use both unique reads and a fractionally assigned multi-mapped reads as an initial estimate of locus-specific expression which subsequently undergoes cycles of E–M calculations until a “convergence” is reached giving an estimate of locus expression. L1EM was built specifically for LINE-1 expression counting and is unique in its explicit modeling of various potential sources of LINE-1 RNA production to improve specificity for bona fide transcripts initiated from the LINE-1 5′UTR promoter [165]. However, it focuses on highly expressed loci by applying a cutoff of at least 100 reads to call expression of a given LINE-1 locus, which could miss out on biologically relevant but moderately expressed loci.

Ultimately, the major limitations of NGS-based mapping of RNA reads to individual TE loci primarily stem from their short read lengths. Although TE copies can be highly similar, they acquire characteristic single nucleotide polymorphisms (SNPs) over time that could be used to distinguish individual loci, provided a sequencing read has sufficient accuracy and length to span the SNPs. Thus, long-read RNA sequencing can, in principle, improve locus-specific mappability (Fig. 5B) [156, 168]. Recently, Berrens et al. demonstrated a proof-of-concept implementation of ONT sequencing for quantifying full-length locus-specific TE expression in single cells, called “CELLO-seq” [169]. CELLO-seq combines a splint oligo ligation step with template-switching reverse transcription to generate full-length cDNAs of polyadenylated transcripts. Template switching is an inherent property of certain viral reverse transcriptases (RT) where upon reaching the capped 5′ termini of mRNAs, the RT exhibits terminal transferase activity, adding overhanging cytosines [169]. In the presence of a guanine-rich forward adaptor, the RT subsequently “switches” template from the mRNA to the adaptor effectively attaching the adaptor sequence to the first strand cDNA 3′ end. A splint oligo ligation step then attaches a 22 base pair unique molecular identifier (UMI) onto the cDNA 5′ end followed by second strand synthesis. The UMI allows for post-sequencing error correction via a machine learning algorithm trained on benchmarked long read datasets [169], the rationale being to overcome the accuracy limitations of long reads for improving mapping of sequenced full-length cDNA molecules to their source TE loci. Notably, the use of the latest ONT R10.4.1, which achieves Q20 + basecalling, will likely further boost the accuracy of sequenced TE RNAs. Importantly, the improvements in error-corrected accuracy and the use of long reads allowed for the first ever detection of allele-specific expression of specific TE loci. Long RNA reads also permitted the identification of full-length TE-exonized chimeric transcripts which frequently occur in cancers, as discussed earlier. Lastly, the use of a template switching mechanism for cDNA synthesis is critical as this captures the information from the 5′ transcription start site per RNA molecule, allowing for discrimination of *bona fide* TE transcription initiated from the TE promoter as opposed to passive intronic readthrough from the host gene (Fig. 5B).

### Profiling TE-associated chromatin and epigenetic marks

TEs contain rich repositories of TF binding sites and thus participate in and are controlled by diverse epigenetic mechanisms. High-throughput sequencing assays have become standard tools for surveying epigenetic landscapes to dissect regulatory mechanisms. Common analyses include measuring DNA methylation, histone modifications, chromatin accessibility, TF occupancy, and 3D genome architecture. Each assay requires dedicated software to process raw sequencing data, perform normalization and statistical inference steps, and visualize results as interpretable readouts. These often do not have dedicated support for TE analyses and require modifications to account for multi-mapping reads. Nevertheless, unlike transcriptomics, the application of chromatin-based sequencing analyses to TEs is generally more straightforward. As most regulatory regions of TE loci are positioned at their termini, such as the 5′UTR promoter of LINE-1, chromatin assays can often leverage flanking unique genomic DNA to anchor reads. Moreover, regulatory sequences of TEs tend to acquire more sequence divergence likely due to greater selective pressures on their regulatory function [170], resulting in increased mappability. However, the internal sequences of repeats, particularly those of the youngest TEs, still complicate most types of chromatin analyses, requiring methods to rescue multi-mapped reads as done with RNA-seq. New assays using long-read sequencing will certainly improve mappability and enable a more complete characterization of the epigenetic landscape spanning entire TE loci. Here, we briefly describe recent advances in TE-centric epigenomic technology development, with a focus on DNA methylation, TF binding, and 3D genome interactions, and discuss promising long-read applications. We also highlight recent uses of CRISPR affinity proteomics for discovering novel chromatin regulators of TE loci.

DNA methylation is a potent epigenetic control mechanism for developmental stage- and tissue-specific gene expression [171]. In humans, DNA methyltransferases (DNMT) catalyze the covalent addition of a methyl group onto cytosine residues (5mC), often at genomic regions with clusters of CpG dinucleotides or so-called “CpG islands”. DNMT3A and DNMT3B are de novo methylators which act on unmethylated cytosines, whereas DNMT1 propagates existing 5mC marks during cell division. Young TEs are prominent targets of DNA methylation, whereas older TEs tend to be primarily regulated by histone modifications [64]. As cancer cells frequently acquire genome-wide hypomethylation during tumorigenesis, resulting in the upregulation of many repeats, DNA methylation is an important proxy for the transcriptional status of a TE locus. NGS-based methods primarily detect 5mC using the unique chemistry of bisulfite conversion. Purified DNA is treated with sodium bisulfite to selectively deaminate unmethylated cytosine into uracil (converts into thymidine during PCR steps) whereas 5mC remains unaltered [172, 173]. Although effective, bisulfite sequencing is generally costly, limiting the achievable throughput across conditions and the resolution at loci of interest within samples. Moreover, bisulfite treatment fragments DNA which in conjunction with the reduced complexity of three-base sequencing reads hinder mappability to repeats. Targeted amplicon sequencing solves this problem by enriching targets of interest. Sanchez-Luque et al. developed a locus-specific bisulfite sequencing strategy to detect the methylation status of specific L1Hs 5′UTR promoters [174, 175]. In this method, L1Hs 5′UTR promoter(s) of interest from bisulfite treated genomic DNA are selectively amplified with primers designed to target the 5′ genomic flank of specific L1Hs loci and ~ 500 bp into the LINE-1 5′UTR (Fig. 5C) [69, 141, 175]. Another method combined the suppression PCR approach for enriching LINE-1 loci from ATLAS-seq with bisulfite sequencing, called “bs-ATLAS-seq”, to detect high-resolution, locus-specific DNA methylation of LINE-1 5′UTR promoters, including non-reference insertions, at genome-wide scale [62]. Although informative, these NGS-based methods still lack the ability to resolve the internal sequences of TEs. To circumvent these limitations, a recent long-read-based strategy to sequence TEs was devised, along with an accompanying software package called “TLDR” for detecting and visualizing 5mC signals from ONT sequencing data [176]. The use of ONT sequencing is a critical feature because the nanopores can directly detect modified nucleotides like 5mC based on their characteristic impact on the electric current in the flow cell as bases translocate through the pores. Moreover, long reads can not only detect methylation along the entire transcription unit but also identify the methylated status of non-reference insertions generated by *bona fide* retrotransposition (Fig. 5C) [176]. The authors demonstrated their proof-of-concept using various normal tissues as well as paired tumor and non-tumor liver samples, revealing an unexpected finding that certain “hot” L1Hs loci previously known to generate retrotransposition in cancers are also hypomethylated in normal liver tissue, which challenges the current model that TE reactivation requires cancer-specific hypomethylation of their promoters [176]. Thus, applications of long reads like TLDR are increasingly revealing the complexities of TE regulation in cancer.

Beyond DNA methylation, diverse epigenetic factors are known to bind to or modify chromatin at regulatory sequences of TEs. In recent years, chromatin immunoprecipitation sequencing (ChIP-seq) has become the de facto standard assay for profiling histones and TF occupancy at loci of interest including TEs. Combinations of histone tail modifications are well-known to demarcate *cis*-regulatory regions; for example, active enhancers are generally marked by H3K4me1 and H3K27ac while active promoters harbor H3K4me3 and H3K27ac, forming the basis for how TEs have been defined as having enhancer or promoter activities in various biological contexts. Like other sequencing analyses, ChIP-seq data for TEs similarly requires consideration of multi-mapping reads. Software packages have been developed using varying strategies to redistribute multi-mapping reads for NGS-based ChIP-seq data [177]. One notable alternative is the “PAtChER” method developed by Taylor et al. which uses 3D genomic interaction information from HiChIP, a type of chromatin assay that profiles long-range DNA interactions anchored by specific chromatin factors, to improve the mappability of ChIP-seq reads to TEs [178]. The authors reasoned that spatial information from HiChIP can aid in reassigning multi-mapped reads by using interaction data from unique read pairs sharing similar 3D contacts. This strategy was able to improve the mappability of ChIP-seq profiles to TE loci as well as increase the number of detected peaks by 5–20% [178]; however, one trade-off is the need to generate parallel genome-wide Hi-C chromosome conformation datasets to normalize for uneven sequencing coverage across genomic bins. In theory, accurate longer reads result in better mappability to repeats. However, ChIP-seq typically benefits from relatively shorter insert sizes to generate narrower “peaks” corresponding to the footprint of TF occupancy, although some histone modification profiles tend to be represented by broader peaks. Thus, long-read sequencing has had fewer adaptations for ChIP-seq type applications so far. Several recent methods [179–181] leverage ONT sequencing to simultaneously map DNA methylation and TF occupancy either using ectopic expression of a TF of interest fused to a DNA adenine methyltransferase (Dam) or antibody-based recruitment of Dam to chromatin. Because adenine DNA methylation does not naturally occur in eukaryotes, DNA-binding profiles can be inferred by the presence of methylated adenines, an alternative to ChIP called “DamID” [182]. The use of direct modified base sequencing improves the specificity of the DamID protocol, which originally detected methylated adenine indirectly through methylation specific RE digestion, but also detects native m5C, allowing for dual readouts of DNA methylation and DNA binding by a TF of interest.

TEs are emerging regulators of 3D genome organization, yet bespoke tools that can handle repeats are still limited. Studies examining the roles of TEs in chromatin conformation mostly reanalyze data from Hi-C experiments [183], which is a widely used high-throughput variant of the chromosome conformation capture (3C) assay [184]. The key steps involved in generating Hi-C libraries are: 1) chemical crosslinking of chromatin in their native states, 2) digestion of crosslinked DNA with frequent-cutting REs, 3) filling in of digested ends with biotinylated dNTPs, 4) ligation of filled-in and proximally associated DNAs, and lastly, 5) purification and downstream library preparation of ligated chimeric DNAs. Subsequent Hi-C data analysis requires paired-end sequencing to infer spatial proximity from discordant read pairs. Given the complicated experimental workflow, Hi-C analytical pipelines require extensive pre-processing, filtering, and statistical inference steps, which calls for significant computing resource; thus, most software primarily work on uniquely mapped reads [185]. For this reason, most studies related to TEs have lower resolution or have focused on older TEs which possess proportionally more unique mappability.

A few software have been introduced specifically tackling the challenge of repetitive DNA mapping in Hi-C datasets. mHiC is a package designed to rescue multi-mapped reads during Hi-C data analysis by using a generative model to predict the best alignment for each multi-mapped read [186]. The software purportedly improved sequencing coverage by up to 20% from reanalysis of existing Hi-C datasets as well as identified new significant interactions involving repetitive genomic regions. Although potentially useful, it is difficult to fully assess its performance without a proper ground truth. HiTea [187] is a package which focuses on discovering non-reference TE insertions from Hi-C datasets by analyzing “clipped” reads, which are split reads containing unmapped portions that are typically trimmed to keep the mapped portion. One disadvantage of the genome-wide nature of Hi-C experiments is the lower resolution, requiring significant sequencing to detect interactions at specific loci. Raviram et al. developed a targeted approach to detect TE-centric interactions by combining hybridization capture of TE subfamilies of interest with Hi-C [188]. Their capture probe design specifically enriches reads that span the genomic junction of the TE targets to improve mappability of read pairs. This junction capture strategy is a cost-effective way to detect higher resolution interaction profiles at TE loci of interest. Moreover, given the repetitive nature of TEs, careful oligo design based on consensus TE sequences may efficiently capture multiple loci at once, enhancing multiplex capabilities. However, capture based 3C experiments notably require special consideration for normalization and statistical significance modeling separate from Hi-C analyses [189]. Lastly, a long-read sequencing based 3C assay called “Pore-C” [190] was developed which leverages the ONT platform to sequence the entire chimeric ligation product generated during 3C library preparation. This approach uniquely detects “multi-way” interactions, which are high-order (3 or more) assemblies of chromatin interactions between distal genomic regions within and/or across chromosomes. Although not explicitly examined, TE interactions will surely benefit from long-read based methods such as Pore-C which can map the entire genomic junction for specific TE loci as well as reveal their potential roles in high-order chromatin structure.

Lastly, we briefly highlight recent uses of CRISPR mediated affinity proteomic methods to discover the complete composition of chromatin associated with TE loci [191, 192]. The premise of CRISPR-based locus-specific affinity proteomics methods is the use of catalytically inactive or “dead” Cas9 (dCas9) coupled with programmable single guide RNAs (sgRNAs) to target the dCas9 complex toward specific loci of interest. These approaches leverage the high-affinity biotin-streptavidin interaction for stringent purification of locus-associated proteomes, either by biotinylating dCas9 via biotin ligases and enriching chromatin co-purifying with dCas9 [191, 193] or by fusing promiscuous biotin ligases directly onto dCas9 and biotinylating nearby proteins for subsequent enrichment (“proximity labeling”) [192]. Briggs et al. adapted a proximity labeling method called “C-BERST” [192] to identify proteins bound to the 5′UTR promoters of young full-length LINE-1 promoters [194]. The authors optimized a set of sgRNAs that target the L1Hs consensus sequence with limited binding of older subfamilies. Comparing the E006AA-hT and LNCaP prostate cancer cell lines, which have low versus high LINE-1 expression, respectively, they identified known LINE-1 associated TFs such as CTCF and YY1, as well as a novel LINE-1 repressor, dual phosphatase 1 (DUSP1). Sun et al. used another variant of proximity labeling called “TurboID” [195] to identify factors bound to primate-specific LTR7/HERV-H in human embryonic stem cells [196], revealing a novel crosstalk between m6A methylation of HERV-H RNAs and DNA methylation of their loci. Specifically, they found that the HERV-H m6A modification binds m6A reader YTHDC2, which subsequently recruits the DNA 5mC demethylase TET1 to maintain expression of LTR7 loci. Because LTR7/HERV-H is an older TE subfamily in the human genome, its sequences are relatively more divergent and higher in copy number. Thus, the authors adapted a multiplex sgRNA cloning strategy called “CARGO” [37, 197] to assemble 15 sgRNAs tiling LTR7 into a single vector. Indeed, the use of CARGO achieved targeting of dCas9 to 1815 copies of LTR7, accounting for 73.5% of all LTR7 loci, as determined by dCas9 ChIP-seq. These two studies showcase complementary sgRNA design strategies that may be considered for future investigations applying CRISPR affinity proteomics for unbiased proteomic discovery of TE chromatin regulators.

## Concluding remarks and perspectives

The molecular geneticist Sydney Brenner famously opined that “progress in science depends on new techniques, new discoveries, and new ideas, probably in that order.” [198] The remarkable trajectory of discoveries in TE biology over the past half-century is testament to this prophetic adage. From Barbara McClintock’s early innovations in cytogenetic techniques, which prompted her discovery of TEs, to present-day breakthroughs in long-read sequencing and multi-omics technologies, our expanding knowledge of the dynamic interplay between TEs and their host genomes has advanced in lockstep with transformative methodology. Now more than ever our molecular tools at hand are revealing the profound, yet still elusive influence of the repetitive fraction of our genomes on cellular health and disease. Many questions remain: Why and how do some cancers reactivate TEs to a greater extent than others? Do some cancers preferentially express certain TE subfamilies? Are there cancer type-specific factors which dictate the “permissivity” of a given TE locus to reactivate? What is the molecular basis of tolerance in epithelial cancers for TE expression whilst evading innate and adaptive immunity? How do LINE-1 gene products contribute to cancer development independent of or in concert with retrotransposition? We speculate that answers to these questions will not only demystify the most abundant entities in the human genome but also unlock new and improved ways to treat cancers.

## Data Availability

There is no data or material to share.
